# An enigmatic soft tissue creeping phenomenon: The spontaneous peri‐implant mucosa margin and papilla growth. A retrospective clinical study

**DOI:** 10.1002/cre2.380

**Published:** 2020-12-21

**Authors:** Ivo Agabiti, Karol Alí Apaza Alccayhuaman, Yasushi Nakajima, Daniele Botticelli

**Affiliations:** ^1^ Pesaro Italy; ^2^ Surgical Division ARDEC Academy Rimini Italy; ^3^ Department of Oral Implantology, Osaka Dental University Osaka Japan

**Keywords:** implant, mucosa growth, peri‐implant mucosa

## Abstract

**Objectives:**

The aim of the present retrospective study was to evaluate papillae filling rate and buccal margins coronal growth in implant‐supported prostheses which were over‐contoured at the apical buccal third to form a rearmost collar zone, thus mimicking a false root.

**Material and methods:**

The influence of adjacent elements, teeth, implants, or pontics was examined. One‐hundred and three crowns have been assessed in photographs taken on 61 patients after prosthesis delivering and at last follow‐up (mean 2.8 years). The Jemt index was adopted to evaluate papillae growth at the baseline and follow‐up as follows: 0, absence of papilla; 1, less than half of interdental embrasure height; 2, more than half of interdental embrasure height; 3, fully papilla filled interdental embrasure. Index score 4, papilla hyperplasia was not included. Moreover, the coronal growth (or recession) of buccal mucosa in implant‐supported crowns was assessed.

**Results:**

At baseline, a total of 29 papillae had a 0 score, while only two scored 3 with complete papilla formation. On follow‐up, only one papilla scored 0, while 46 scored 3 with complete interdental embrasures filling. The highest papilla score was registered from first year crown insertion and likewise in interdental embrasures located between two implants or implants and pontics. Moreover, the buccal margin growth was observed in almost 80% of crowns. Such findings seem to indicate that buccal margin and papilla around implant‐supported crowns presented a coronal growth over time, especially during its first year. The average papilla growth between two implants was no lower than that observed in papilla present between implants and natural teeth.

**Conclusions:**

Recessed areas at collar of implant‐supported prostheses appear to positively influence papillae and buccal margin growth, especially in its first year. Papilla growth between two implants was similar to that observed between implants and natural teeth.

## 1 BACKGROUND

Papillae interproximal space fillings have been described after teeth approximation during orthodontic movement in dental‐arch alignment (Kim et al., 2014; Morris, 1958). A similar phenomenon was also described after implant‐supported crown insertion (Jemt, 1997; Schropp et al., 2005; Schropp & Isidor, 2015). An index to assess interdental papilla growth in implants‐supported single crown has been introduced and it was originally classified into five levels (Jemt, 1997). The evaluation was scored as follows: 0 in the case of papilla absence; 1 when papilla filled less than half and 2 when more than half of interdental embrasure respectively; 3 when interproximal space filling was complete; 4 in the case of overgrowth. The Jemt index had already been used in several researches, proving beneficial to papilla growth study (Cardaropoli et al., 2006;Schropp et al., 2005; Schropp & Isidor, 2015). In one investigation the level 4 was not adopted (Schropp et al., 2005; Schropp & Isidor, 2015). Indeed, level 4 cannot be interpreted as an improvement on score 3. On the contrary, it can represent prosthesis conformation errors, plaque accumulation, or drug assumption which can influence keratinized mucosa overgrowth. Spontaneous papillae growth within interproximal spaces have been investigated and various causes of this phenomenon were suggested. Main inducing factors, as alveolar crests buccal‐lingual width (Chang & Wennström, 2013), distance between two elements, both in teeth or implants (Kim et al., 2014; Tarnow et al., 2000; Tarnow et al., 2003), contact point position with adjacent elements, were all cited (Choquet et al., 2001; Tarnow et al., 2003). Moreover, an inflammatory theory was advocated as cause of papillae growth (Jemt, 1997). A recent hypothesis indicating intraoral negative pressure of swallowing was proposed as cause for oral mucosa growth (Harster, 2005; Santander et al., 2013). In a clinical study, the negative pressure was measured in two main compartments of the oral cavity: the sub‐palatal space (SPS) and that of the inter‐occlusal space (IOS). The latter is located within teeth proximity, enclosed between cheeks/lips and tongue, involving the whole arch. After swallowing, an average compartment negative pressure of almost −50 mbar was measured, stably maintained with lips closed until the next reopening. This negative pressure that would enable soft tissue sucking, also might explain the “linea alba” formation (Takagi & Sakurai, 2003) and probably that of tongue indentation which it is often associated with (Piquero et al., 1999; Yanagisawa et al., 2007). This mechanism could be at the base of periimplant keratinized tissue growth. One must take into consideration, as resulted in a research that any negative pressure of swallowing can cause shifting of soft tissues within empty spaces (Harster, 2005). Some studies also assessed long term buccal soft tissue margin modifications which had a retracting tendency rather than growth (Cardaropoli et al., 2006; Jemt, 1997). Further studies have assessed papilla and marginal soft tissue growth around single crowns, concentrated on contact point height (Cardaropoli et al., 2006; Jemt, 1997; Tarnow et al., 2003). However, the whole crown anatomy may have a significant soft tissue management impact on margins and papillae (Kim et al., 2014). When no contact point between two teeth occurs as in diastema no papilla is detected, given that negative pressure cannot be created and maintained. Mucosa structures wall lining form empty compartments around the crowns, hence swallowing might create a negative pressure environment (Engelke et al., 2011), as seen in interproximal and collar recessed spaces on the buccal aspect. An over‐contoured prosthetic crown creates a recessed zone increase at the collar (false root), which might influence thickness and height in soft tissues growth. The so called “inaccessible surfaces” of dental arches are unreachable by muscular action of lip, cheek and tongue (Morris, 1958; Morris, 1962). Mucosa walls structures, when resting on crowns, form empty compartments, where swallowing creates an environment of negative pressure (Engelke et al., 2011) in both interproximal spaces and in buccal recessed spaces at collar. An over‐contoured prosthetic crown at apical third creates as consequence a recessed zone at collar (false root), which can influence soft tissues thickness and height. Ovate pontics in implant‐supported prosthesis, could positively influence the filling of interdental spaces by papillae as well (Calesini et al., 2008).

To our knowledge, no evidence in soft tissue growth at recessed collar zones has been shown yet. Hence, the aim of the present retrospective study was to evaluate papillae filling rate and buccal margins coronal growth in implant‐supported prostheses which were over‐contoured at the apical buccal third to form a rearmost collar zone, thus mimicking a false root. A three‐dimensional closed compartment via cheeks and lips was formed (as in interdental embrasures). The influence of adjacent elements; teeth, implants or pontics, was also considered to assess soft tissue growth phenomenon.

## 2 MATERIALS AND METHOD

In this retrospective clinical study, patients were selected from one of the author's private dental office archives (IA). The study was performed following the declaration of Helsinki on medical protocols and ethics. Informed consent was signed from all patients. The inclusion criteria were the following: (i) photograph availability, taken at prosthesis delivering and follow‐up; (ii) papilla index score evaluation possibility; (iii) a 3 months' minimal follow‐up. The exclusion criteria were: (i) the absence of both initial and follow‐up photographs; (ii) impossibility to evaluate the index score in photographs; (iii) signs of peri‐implantitis at follow‐up; (iv) use of temporary fixed bridge before final restoration. The papillary score used in the present study was based on that established by Jemt (1997). The index was evaluated in photographs taken within 2 weeks from prosthesis delivering and at follow‐up. Four of the five Jemt's index scores were adopted, based on the closure of interdental papilla embrasure: 0, absence of papilla; 1, less than half of interdental embrasure height; 2, more than half of interdental embrasure height; 3, fully papilla filled interdental embrasure. Index score 4, papilla hyperplasia was not included (Schropp et al., 2005). The score measurement by Jemt index, implant‐supported crown papilla base was used. Moreover, coronal growth (or recession) of buccal mucosa in implant‐supported crowns was also assessed. In order to perform margin measurement, mucosa level of adjacent teeth or that of collar stained margin on crown surface, were used as reference. The following criteria were adopted: ++, clinically relevant coronal growth; +, moderate coronal growth; 0, no growth, no recession; −, moderate recession; −−, clinically relevant recession. Implants with three different geometric conformation produced by same company were used (Pilot, Kohno and Premium implants; Sweden & Martina—Due Carrare, PD, Italy). In most delayed cases, a splitting ridge technique was used. After implant installation, split‐thickness flaps apically repositioned were adapted around the healing abutment, allowing a not‐submerged healing. A flapless procedure was used in immediate implant insertion after tooth extraction. No removable or implant supported temporary prosthesis were applied. After 3–4 months healing, impressions were taken. Chamfered shoulder titanium customized abutments were screwed onto the implants and porcelain single crowns or bridges on gold or zirconia were cemented. The prosthetic crowns and pontics were over‐contoured at the buccal apical third, creating an under‐contoured area at the collar, mimicking a false root. All pontics were ovate shaped and most crestal mucosa prepared with a trapezoidal incision following the “edentulous site enhancement” technique (Calesini et al., 2008). Split‐thickness flaps were dissected, and a collagen sponge was placed between wound and ovate pontic. Photographs were taken within 2 weeks the prosthesis delivering. After fixed prosthesis placement, patients were instructed to perform an accurate oral hygiene practice and were invited to follow a recall appointment at 6‐month intervals.

### 2.1 Data analysis

Mesial and distal aspects were considered separately. The number of papillae classified in each index were evaluated at the insertion of prosthesis and at follow‐up. Evaluations were performed independently by two examiners (IA and KAAA). Any disagreements between the two assessors were solved by a consensus with a third author (DB). Moreover, similar evaluations were also performed after categorization in adjacent elements to the implant (tooth, implant, or pontic), timing of follow‐up, genders, and type of restoration. A mean index was used (Jemt, 1997). The Wilcoxon test was applied for statistic evaluations between data of baseline and follow‐up. For explorative purposes, differences between genders and age groups ≤35 years and ≥70 years were statistically evaluated using the Mann–Whitney *U* test.

## 3 RESULTS

**FIGURE 1:**
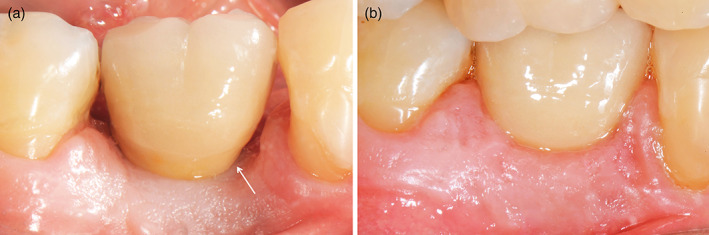
Buccal view of implant‐supported single crown in 4.6 position. (a) At prosthesis delivering period; (b) at 1 year follow‐up. Interproximal embrasures were completely papillae filled; also, buccal tissue growth until false root limit. The white arrow points to papilla base

**FIGURE 2:**
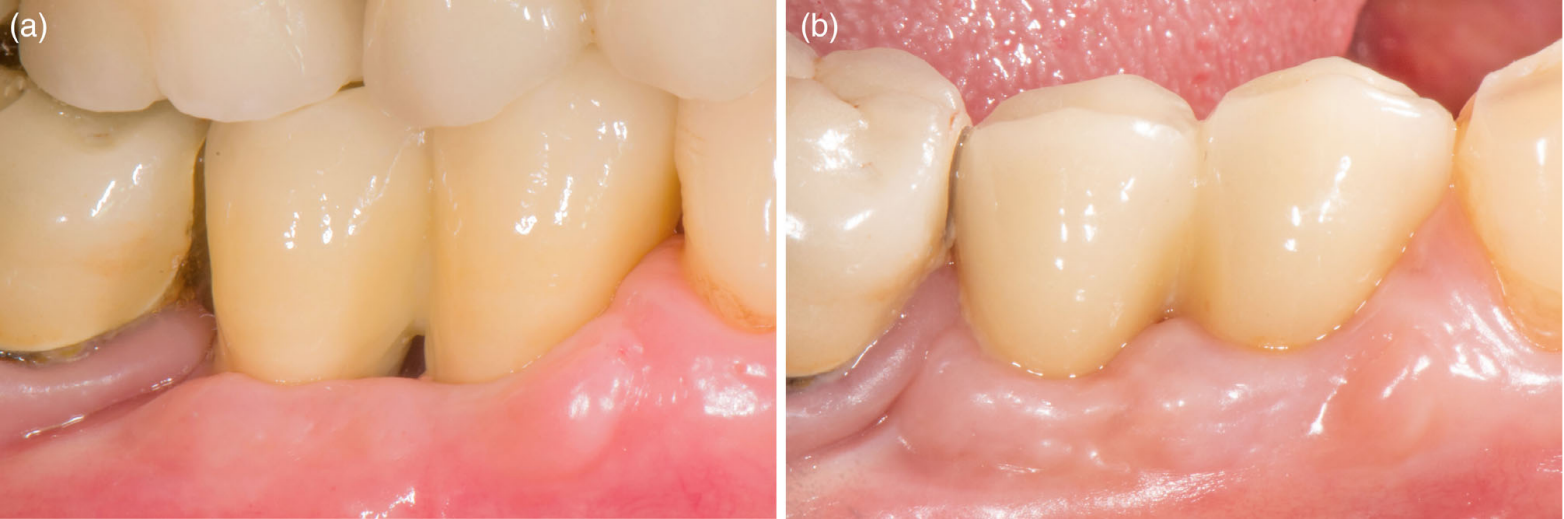
Buccal view of two implant‐supported splinted crowns in 4.5–4.6 position. (a) At prosthesis delivering period; (b) at 6‐year follow‐up. Inter‐implant embrasure was completely papilla filled, as well as embrasures between implants and natural teeth; papillae grew over previous level also on natural teeth side, covering distal crown gold margin. An important tissue margin coronal growth was evident

**FIGURE 3:**
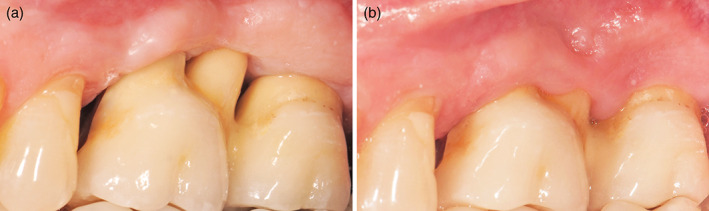
Buccal view of two implant‐supported splinted crowns in 2.6–2.7 position, with ovate pontic as distal false root in 2.6. (a) At prosthesis delivering period; (b) at 8.5 years follow up. The two false root interproximal embrasures were completely papillae filled. Note important marginal soft tissue growth

**FIGURE 4:**
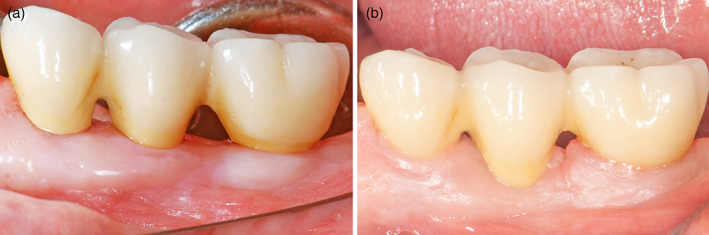
Buccal view of two implant‐supported 3‐unit bridge in 34–36 position and with ovate pontic in between. (a) At prosthesis delivering period. (b) At 4 years follow‐up. Pontic embrasures were partially filled by papillae. Note: Significant two implants marginal soft tissue growth, along with a small recession at pontic was detected, probably due to excessive ovate pontic collar dimension

**FIGURE 5:**
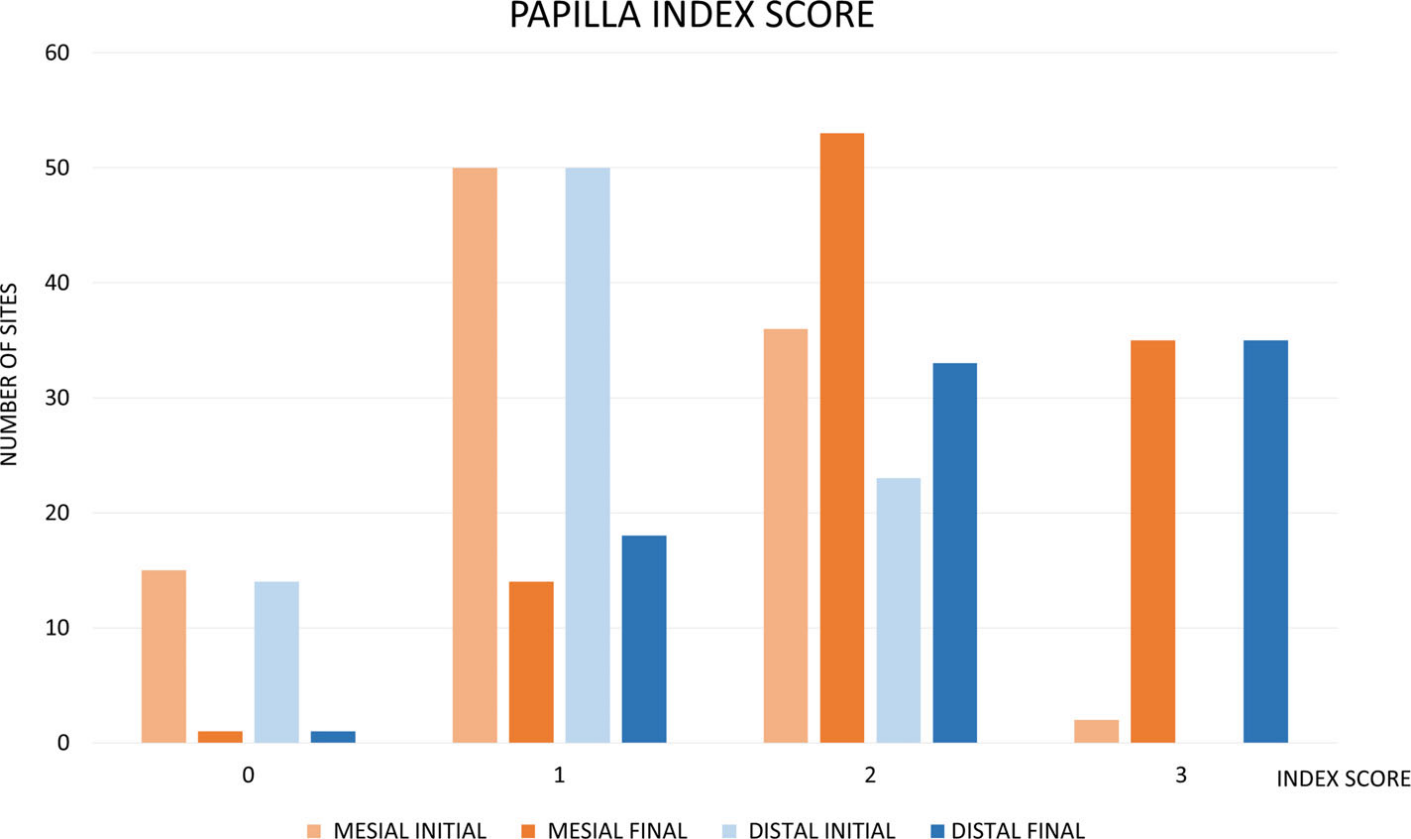
Graph illustrating sites number in mesial and distal sides at prosthesis delivering and follow‐up. Jemt index score 0, absence of papilla; 1, less than half in interdental embrasure height; 2, more than half in interdental embrasure height; 3, interdental embrasure completely filled by papilla

With present study on a total of 82 patients from initial to follow‐up photographs were evaluated. At prosthesis delivering, papillae were still not formed, so, primitive papillae level in mesial and distal sides, were located at different level compared to adjacent elements (Figure 1). A possible papillary index assessment of 103 implants is performed on 61 patients. Thirty‐seven females, with a mean age of 51.9 ± 13.8 years and 24 males, with a mean age of 55.2 ± 14.5 years were included (Table 1). Seventy‐five different prosthetic structures were provided, of which 51 were single crowns (Figure 1), eight were splinted crowns supported by two implants (Figures 2 and 3), 11 were 3‐units bridges with two implants and the pontic in a central position (Figure 4), and five were 4‐units bridges supported by two or three implants. The mean period of follow‐up was 2.8 years. In detail, 32 implants had a follow‐up ≤1 year, 40 implants >1 year to 3 years, and 31 implants >3 years. Implants position was molar region in 54 cases, premolars region in 38 cases, and frontal maxillary region in 11 cases. Considering both mesial and distal sides at initial stage, 29 papillae were scored as index 0, 100 as 1, 59 as 2 and only two as 3 (Figure 5). At follow‐up, one papilla was scored as index 0, 27 as 1, 56 as 2 and 46 as 3. The peri‐implant buccal margin mucosa presented a moderate coronal growth in 45 crowns and a clinically relevant coronal growth in 35 cases. Twenty‐three implant sites did not present any growth while no crowns had recession at buccal aspect. Mean values of papilla index score in initial stage was 1.2 ± 0.7 at mesial aspect, and 1.1 ± 0.6 at distal aspect. At final stage, papilla index score was 2.2 ± 0.7 at mesial aspect, and 2.2 ± 0.8 at distal aspect with a mean score gain 0.9 ± 0.8 and 1.1 ± 0.8, respectively. The difference between the two periods was statistically significant for both the mesial and distal aspects (Table 2). Considering mesial and distal aspect mean score in 103 implants was 1.0 ± 0.7 (referred to implants), while in 61 patients was 1.0 ± 0.5 (referred to patient). Mesial side adjacent elements of which 74 were natural teeth, implants in 15, and pontics in 14 (Table 1). At adjacent distal side, were 60 times teeth, 11 times implants, 16 times pontics, and 16 times a final element. At prosthesis delivering, mean papilla index score was 1.4 ± 0.7 for adjacent teeth, 1.1 ± 0.9 for implants, and 0.8 ± 0.7 for pontics. A mean gain at follow‐up was 0.9 ± 0.7, 1.5 ± 1.0, and 1.4 ± 0.8, respectively. All the differences were statistically significant (Table 3). Mean gain in various periods at mesial and distal sides was 0.8 ± 0.7 and 1.1 ± 0.7 at 1‐year follow‐up, 1.0 ± 0.8 and 1.0 ± 0.8 between >1 year and 3 years, and 1.0 ± 1.0 and 1.2 ± 0.9 at follow‐up >3 years, respectively. All the differences were statistically significant (Table 4). Females presented an initial mean papilla index score of 1.4 ± 0.8 mesially, and 1.2 ± 0.6 distally, with a gain after a mean 3‐year follow‐up of 1.0 ± 0.8 mesially, and 1.1 ± 0.8 distally. In males, initial mean papilla index score was 1.1 ± 0.8 mesially, and 0.9 ± 0.6 distally, with a gain after a mean follow‐up at 2.6 years of 0.9 ± 0.9 and 1.0 ± 0.8, respectively. No statistically significant differences were disclosed in score gain between genders both at the mesial (*p* = 0.812) and distal (*p* = 0.653) aspects. The highest score gain was registered in younger patients compared to older ones. In patients ≤35 years, the index score gain was 1.3 ± 0.8 and 1.5 ± 0.5 at the mesial and distal aspects, respectively. The lowest gain scores were observed in the older patients, with gain of 0.9 ± 0.5 at the mesial aspect, and 0.6 ± 0.8 at the distal aspect (p = 0.091 and p = 0.012 respectively between the two age groups).

**TABLE 1:** Demographic data

Gender	Age	Element substituted by implants	Prostheses	Follow‐up	Adjacent element mesial	Adjacent element distal
37 females 24 males	Females 51.9 ± 13.8 Males 55.2 ± 14.5	Molars: maxilla 31; mandible 23 Premolars: maxilla 26; mandible 12 3 Canine; 8 incisors maxilla	51 Single 8 Splinted crowns 3‐ and 4‐units bridges 16	≤ 1 year 32 implants >1–2 years 20 implants >2–3 years 20 implants >3–4 years 11 implants >4 years 20 implants	74 teeth 15 implants 14 pontics	60 teeth 11 implants 16 pontics 16 last elements

**TABLE 2:** Mean index scores ± standard deviation of the full set of data (SD)

	Mesial				Distal			
	Insertion	Follow‐up	Gain	*p*‐Value	Insertion	Follow‐up	Gain	*p*‐Value
Mean ± SD	1.2 ± 0.7	2.2 ± 0.7	0.9 ± 0.8	<0.000	1.1 ± 0.6	2.2 ± 0.8	1.1 ± 0.8	<0.000

**TABLE 3:** Mean index scores ± standard deviation of papillae based of the adjacent element, that was a tooth, an implant, or a pontic

	Mesial				Distal			
	Insertion	Follow‐up	Gain	*p*‐Value	Insertion	Follow‐up	Gain	*p*‐Value
Teeth	1.4 ± 0.7	2.1 ± 0.7	0.8 ± 0.8	<0.000	1.2 ± 0.6	2.1 ± 0.8	0.9 ± 0.7	<0.000
Implants	1.1 ± 0.9	2.5 ± 0.7	1.3 ± 0.9	0.001	1.1 ± 0.8	2.5 ± 0.7	1.5 ± 1.0	0.007
Pontics	0.8 ± 0.7	2.1 ± 0.8	1.3 ± 1.0	0.003	0.9 ± 0.7	2.3 ± 0.7	1.4 ± 0.8	0.001

**TABLE 4:** Mean index scores ± standard deviation of papillae based of the period of follow‐up

	Mesial				Distal			
	Insertion	Follow‐up	Gain	*p*‐Value	Insertion	Follow‐up	Gain	*p*‐Value
≤1 year	1.3 ± 0.7	2.2 ± 0.7	0.8 ± 0.7	<0.000	1.2 ± 0.6	2.3 ± 0.7	1.1 ± 0.7	<0.000
>1 to ≤3 years	1.2 ± 0.7	2.1 ± 0.8	1.0 ± 0.8	<0.000	1.1 ± 0.7	2.1 ± 0.8	1.0 ± 0.8	<0.000
>3 years	1.3 ± 0.9	2.3 ± 0.6	1.0 ± 1.0	<0.000	1.0 ± 0.6	2.2 ± 0.9	1.2 ± 0.9	<0.000

The mean gain at the mesial and distal aspects for various typologies of prostheses were 0.9 ± 0.7 and 1.0 ± 0.7 for single tooth, 0.9 ± 0.9 and 1.1 ± 0.9 for 3‐ and 4‐units bridges, and 1.0 ± 1.0 and 1.4 ± 1.0 for 2‐units splinted crowns. No differences were disclosed among the various types of prostheses.

## 4 DISCUSSION

The present study aim was to evaluate papillae filling rate and buccal margins coronal growth in implant‐supported prostheses which were over‐contoured at apical buccal third to form a rearmost collar zone, thus mimicking a false root. A three‐dimensional closed compartment via cheeks and lips was formed (as in interdental embrasures). Papillae filling and buccal soft tissue margin height increased spontaneously overtime in most of the prostheses evaluated. Peri‐implant soft tissues have progressively filled both interdental embrasures empty spaces and recessed collar spaces, adapting to the shape of underlying prosthetic structures. Soft tissues grew spontaneously between crowns of implant‐supported prostheses taking on similar appearance to natural papillae. At initial stage, 29 papillae presented Jemt index score 0, while only two had scored 3. At final stage, only one papilla had scored 0, while 46 presented a score 3, resulting in interdental embrasures completely filled with papilla. In implant‐supported crowns, papilla growth has been demonstrated in various studies (Cardaropoli et al., 2006; Jemt, 1997; Priest, 2003;Schropp et al., 2005; Schropp & Isidor, 2015). In a retrospective analysis (Jemt, 1997), 25 single crown photographs taken at prosthesis delivering and after a mean follow‐up at 1.5 years were evaluated by Jemt index. Mean scores were 1.44 and 1.52 at mesial and distal papillae, respectively. At follow‐up, a gain of almost 1 score value was obtained on both sides. This result is similar to that observed in the present study.

In a randomized controlled trial (Schropp et al., 2005; Schropp & Isidor, 2015), the papilla score was evaluated after 1.5, 5, and 10 years after prosthesis delivering. After 10 years, it was shown that one third of papilla had completely filled the interproximal space and less than 60% presented an adequate clinical crown length. These results are similar to that observed in the current study where the papilla filled the interdental embrasures in almost 37% of cases. It was also observed that 80 out of 103 crowns presented a buccal margin coronal growth, in which 35 of these was clinically relevant.

In a prospective clinical study including 11 patients (Cardaropoli et al., 2006), 1‐year after single crown prosthesis delivering, 86% papillae were occupying equal to or greater than 50% interproximal spaces, and 18% cases a complete papilla filling was observed. However, at buccal side, 0.6 mm mean marginal soft tissue recession was registered. The papilla filling outcome was similar to that of the present study in which a proportion of 82% was observed with index score ≥ 2. However, no recessions were registered.

In a retrospective study on single implant restoration (Priest, 2003) after a mean follow‐up of 3.5 years, a complete papilla filling was observed in 75% of 55 implants examined. Nevertheless, buccal soft tissue marginal level remained quite stable over time. These outcomes are not completely in agreement with those of present study in which almost 37% of the interdental spaces were filled by papilla, and only 22% did not show any soft tissue marginal growth.

The present study revealed that most papilla score improvement occurred during its first year, while, subsequently, only a slight increase was observed. This is in agreement with a previously discussed study in which the highest gain was observed at 1.5 years follow‐up (Schropp & Isidor, 2015).

Moreover, even though fewer implants (17) were assessed in the first 6 months' period, a similar to 1‐year main papilla score gain was observed. Consequently, papilla growth seems to occur during the first stage healing period.

In the present study, mesial or distal adjacent elements to implant‐supported crowns were 134 times natural teeth, 26 times implants and 30 times pontics. Sixteen times distal sites were a final element and not included in papilla evaluation. Moreover, data showed a highest score if adjacent element was of another implant or pontic, respectively. All pontics were ovate shaped and in most the “edentulous site enhancement” technique was applied (Calesini et al., 2008).

The lowest papilla score was observed when a natural tooth was adjacent to the implant‐supported crown. It might be argued that initially, the papilla was higher if a tooth was adjacent (mean score 1.4) compared to an implant (mean score 1.1), a fact that might reduce the option of papilla growth. Nevertheless, the final score was 2.1 for adjacent tooth, and 2.5 for adjacent implant.

However, it is to be considered that in cases of two implant‐supported splinted crowns or pontics, the contact point was more apically positioned due to the bigger dimensions of the connector which reduced the height of the embrasure favoring papilla filling (Tarnow et al., 2003).

This might explain why the mean score was slightly higher at the 3‐ and 4‐units bridges and two implant‐supported splinted crowns compared to the single crowns.

The importance of the contact point distance to the bone crest has been clinically evaluated in natural teeth (Cardaropoli et al., 2006; Choquet et al., 2001; Tarnow et al., 2003). It was reported that a distance equal to or less than 5 mm allows a filling of the interproximal space in almost 100% cases, while percentage diminishes progressively if the distance increase (Choquet et al., 2001). Nevertheless, it has been shown that also single crowns with more than 9 mm distances between contact point and bone crest, might have a complete papilla filling (Grunder, 2000). Moreover, it was demonstrated that mucosal height might be influenced by given distance between implants and recommended to be of at least 3 mm to reduce bone marginal resorption.

In the current study, papilla growth was observed at all ages. Nevertheless, higher mean scores were seen in younger patients (≤35 years) compared to older ones (≥70 years). This is in compliance with another study where patients over 50 years of age scored better compared to those <50 (Schropp & Isidor, 2015).

Ridge splitting techniques and apically repositioned flaps were applied in several cases in the present study, aiming to increase the edentulous crests width and consequently the keratinized mucosa. Moreover, it has been shown that papillae height is positively correlated with the buccolingual crest width (Chang & Wennström, 2013).

Several conditions have been described to influence papillae growth. However, the mechanisms have not been clearly disclosed yet. It was suggested that the spontaneous process is influenced by the alveolar crest buccal‐lingual width (Chang & Wennström, 2013), or the distance between teeth (Kim et al., 2014) and implants (Tarnow et al., 2000; Tarnow et al., 2003) or, the crest bone to contact point distance (Choquet et al., 2001; Tarnow et al., 2003). The latter has been questioned by a systematic review, disclosing limited evidence to support this hypothesis (Roccuzzo et al., 2018). Another author suggested that inflammation and swelling subsequent to plaque accumulation in interproximal regions might be the first step of papilla growth. Inflamed tissue subsequently turns into mature papilla over time (Jemt, 1997). However, histological analysis on keratinized tissues which had progressively filled all empty spaces formed by prosthesis, failed to detect inflammatory infiltration, found only in close contact abutments surfaces and prosthetic structures (Harster, 2005).

Another considerable factor in soft tissue growth mechanism is the negative pressure established in oral cavity due to swallowing (Harster, 2005).

In a report, mucosal growth underneath overdentures bars was explained as a closed compartment formation with “negative pressure gradient” in dead space (empty) which is formed underneath the bar after prosthesis wearing (Payne et al., 2001). Dead spaces beneath bars are progressively filled by soft tissues in the presence of a good peripheral seal thus ensuring negative pressure development and maintenance (Payne et al., 2001).

As already known, relative intraoral negative pressure is formed during swallowing which mechanism was compared to an “oral pump” (Engelke et al., 2011; Harster, 2005). At rest position, “tongue repositioning maneuver” after swallowing, that is, tongue on the palate, closed lips and nasal breathing, intraoral negative pressure in specific spaces is constantly long‐term maintained—at the same level allegedly during chewing, too. A negative pressure measured with a manometer within interocclusal and interdental spaces is formed between tongue and lips or cheeks which act as closed biofunctional compartments (Engelke et al., 2011; Fränkel, 1980). Some authors affirmed that a negative pressure purposely influences the state of oral soft tissues and/or to modify its shape. In fact, removable and fixed prostheses create small empty suction chambers which stimulate gingival mucosa inward growth (Fränkel, 1980; Harster, 2005).

**FIGURE 6:**
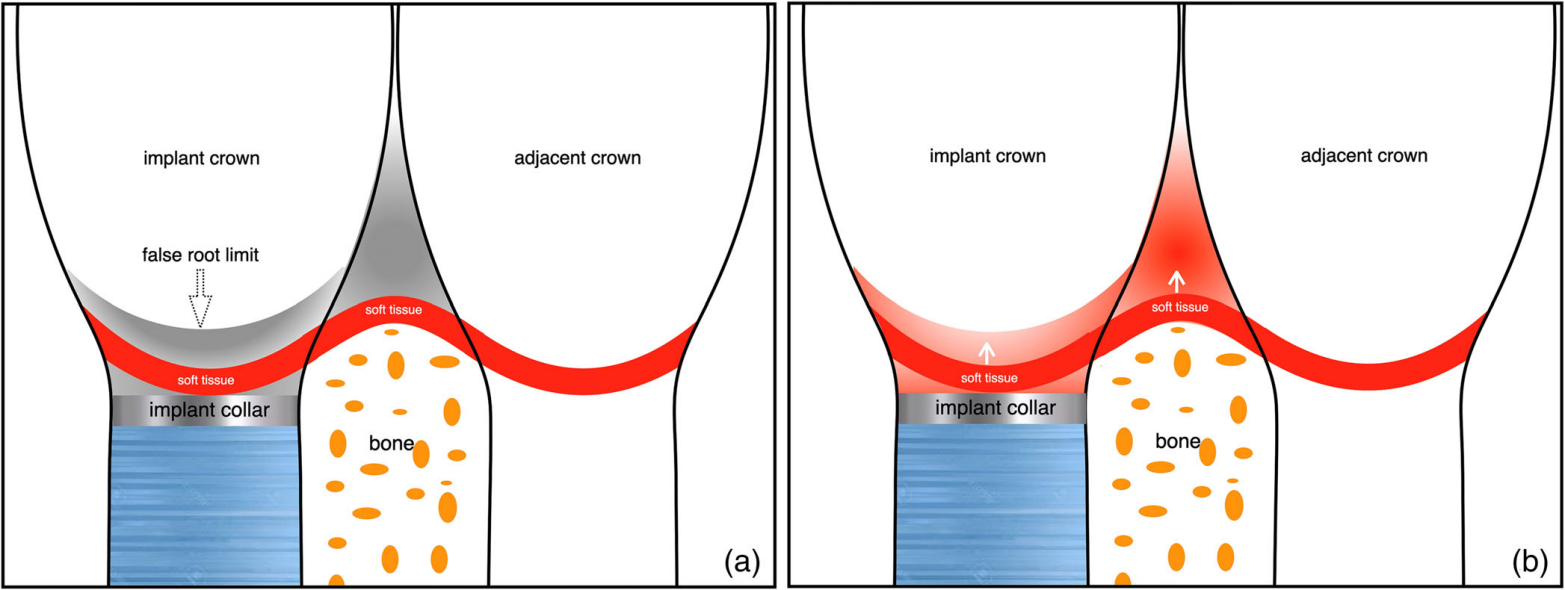
Schematic drawings indicating: (a) interproximal space and collar recessed zones (gray); b) soft tissue growth recessed zones (light red)

It was ascertained that “inaccessible surfaces” have already been defined as unattainable areas around teeth by muscles of lips, cheeks, or tongue (Morris, 1962). Unquestionably, these structures when functional become 3‐dimensional and are more complex to mere surfaces so that the term “spaces” seem more appropriate. Convexities at the apical third of prosthetic crowns at buccal aspect, delimit a more recessed area at the collar. These zones, further to interproximal spaces, are all considered “inaccessible spaces” (Figure 6) (Morris, 1962).

**FIGURE 7:**
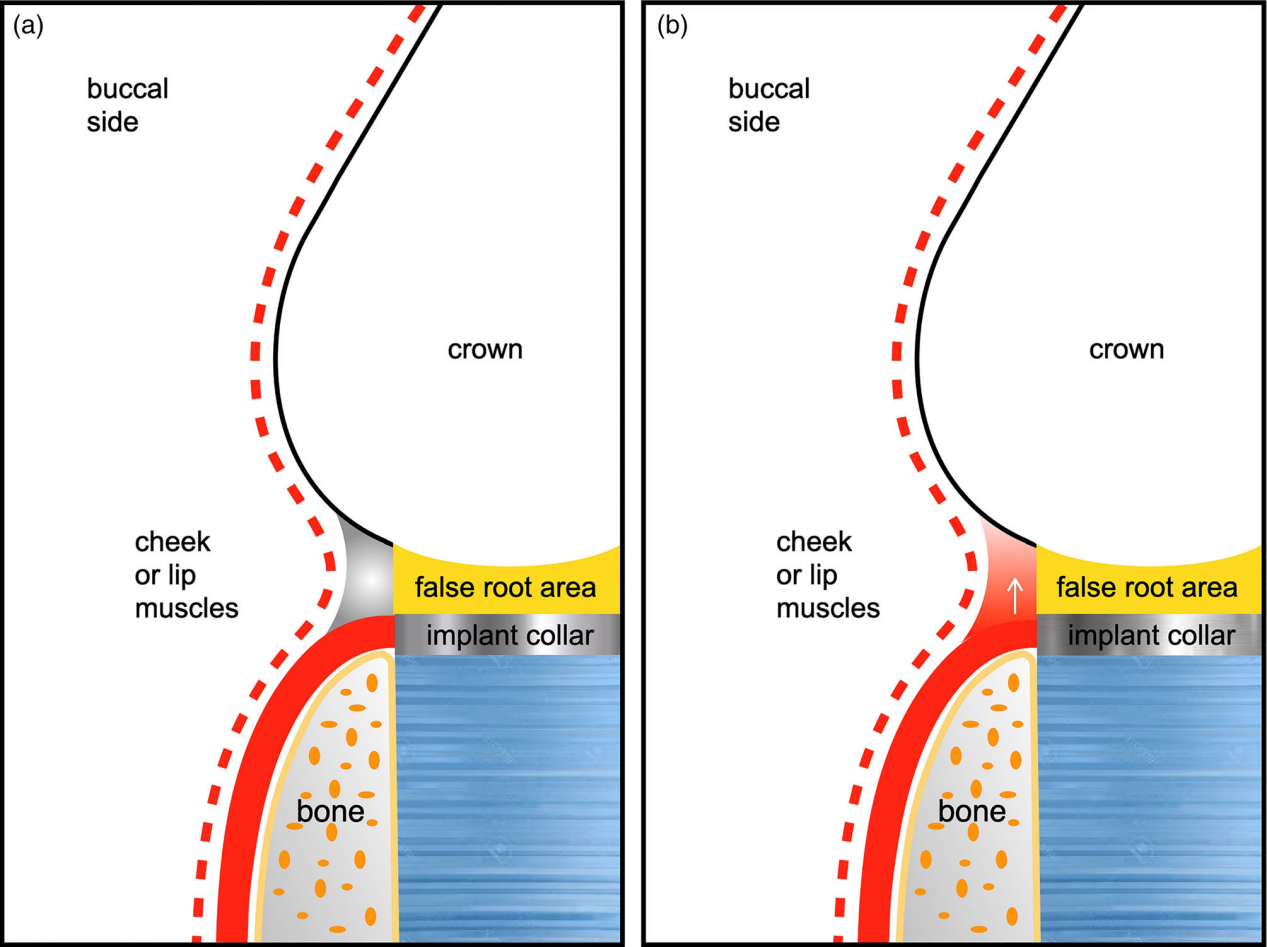
Schematic drawings (Morris, 1958 modified) indicating: (a) buccal aspect recessed zones at collar (gray); (b) soft tissue growth in recessed zones (white arrow). Recessed zones are spaces delimited by mucosa of lips and cheek and by hard crown surfaces. Keratinized soft tissue growth in empty spaces indicated

Reportedly, negative pressure of swallowing, increases keratinized mucosa volume being the only firm tissue that can be sucked into recessed areas, maintaining its shape over time. These enclosed spaces are formed and sealed by cheeks, lips and tongue, acting as small suction chambers around the crown collar and within the interdental spaces (Figure 7) (Harster, 2005).

Prosthetic crowns and pontics in the present study were made following an over‐contouring profile at the buccal apical third creating an under‐contoured recessed area at the collar, mimicking a false root (Figure 7). This was aimed to allow a buccal soft tissue margin coronal growth. This might explain the differences to other studies, reporting a high recession incidence or non‐growth (Cardaropoli et al., 2006; Priest, 2003).

Present study limitations include an aforementioned retrospective design and low number of spaces between two implants, thus limiting conclusive strength. Moreover, the photographs of the baseline and follow‐up were not taken with the same shooting angle. However, this angle deviation between photographs was ≤10° that have been shown to create minor distortions for clinically evaluations (Bertl et al., 2019). Finally, randomized clinical trials comparing crowns with and without recessed at the collar spaces should be performed to disclose differences in papilla and buccal mucosa growth.

Conclusively, recessed areas at collar of implant‐supported prostheses appear to positively influence papillae and buccal margin growth, especially in its first year. Papilla growth between two implants was similar to that observed between implants and natural teeth.

## CONFLICT OF INTEREST

The authors have stated explicitly that there are no conflicts of interest in connection with this article.

## Data Availability

The data that support the findings of this study are available from the corresponding author upon reasonable request.
